# Intraguild Predation of *Hippodamia variegata* on Aphid Mummies in Cotton Field

**DOI:** 10.3390/insects14010081

**Published:** 2023-01-13

**Authors:** Shuying Dou, Bing Liu, Yangtian Liu, Jianping Zhang, Yanhui Lu

**Affiliations:** 1 Xinjiang Production and Construction Corps Key Laboratory of Special Fruits and Vegetables Cultivation Physiology and Germplasm Resources Utilization/Key Laboratory at Universities of Xinjiang Uygur Autonomous Region for Oasis Agricultural Pest Management and Plant Protection Resources Utilization, College of Agriculture, Shihezi University, Shihezi 832003, China; shuyingdou@163.com; 2 State Key Laboratory for Biology of Plant Diseases and Insect Pests, Institute of Plant Protection, Chinese Academy of Agricultural Sciences, Beijing 100193, China; liubing1945@126.com (B.L.); yangtianlwmt@163.com (Y.L.); 3 Western Agricultural Research Center, Chinese Academy of Agricultural Sciences, Changji 831100, China

**Keywords:** predatory ladybeetle, parasitoid, molecular detection, food web, intraguild predation

## Abstract

**Simple Summary:**

When there are multiple natural enemies in the same ecosystem, intraguild predation between natural enemies will affect the population dynamics of target organisms and the effectiveness of biological control. It is necessary to study this relationship in depth to enhance the function of biological control. *Hippodamia variegata* (Goeze) and parasitoids are two types of dominant natural enemies of the cotton aphid *Aphis gossypii* Glover in Xinjiang, China. Among them, the ladybeetle preys on common aphids as well as parasitoids. This study measured the feeding choices of *H. variegata* towards mummies with different densities of *A. gossypii*. Meanwhile, investigation and sampling in the cotton field were conducted from 2017 to 2019. The predation of *H. variegata* individuals on aphids and mummies was detected using diagnostic PCR. The results showed that *H. variegata* had no obvious feeding preference towards live aphids and mummies, and preferred denser prey. The molecular detection results show that A. gossypii was the main prey source and medium of consumption of parasitoids for the ladybeetle. *H. variegata* had strong trophic links to both parasitoids and aphids. The above information is crucial for evaluating the pest control ability of *H. variegata* more comprehensively and strengthening the strategies for the biological control of aphids.

**Abstract:**

Intraguild predation among arthropod predators in agricultural ecosystems may have a negative impact on biological control. At present, there are few direct reports on trophic relationships among participants of predation in field groups. In this study, we measured the feeding choices of *Hippodamia variegata* (Goeze) towards mummies with different densities of *Aphis gossypii* Glover. The dynamics of the occurrence of mummies in the cotton field were investigated over 2017–2019. Singleplex PCR and multiplex PCR were used to detect the predation of 2090 *H. variegata* individuals on aphids and mummies in Xinjiang cotton field, which revealed the intraguild predation frequency between *H. variegata* and various parasitoids. There was no obvious feeding preference of *H. variegata* towards live aphids or mummies, which mainly depended on the relative density of prey. Among the four species of aphids detected in *H. variegata*, *A. gossypii* had a high detection rate and was the main prey source of the ladybeetle in the cotton filed. Mostly, ladybeetles consumed parasitoids through mummies, with 6.39% directly feeding on adult parasitoids. *H. variegata* had strong trophic links to both parasitoids and aphids. We established a food web of aphids–primary parasitoids–hyperparasitoids–*H. variegata*, which can be used to evaluate the pest control ability of *H. variegata* from a new perspective.

## 1. Introduction

Intraguild predation (IGP), which exists widely in ecosystems, refers to the existence of both competition and predation or parasitism among species of the same trophic level, and can be understood as the mutual predation and parasitism among different species of natural enemies of target pests in an agricultural ecosystem [1,2,3]. IGP is common between generalist predators and parasitoids that share common prey. Parasitoids lay eggs in the host pests, and predators usually prey on the parasitoids developing in the host pests [4,5,6]. Such asymmetric IGP may negatively affect the population and dynamics of parasitoids, and even the biological control of pests. Therefore, the study of the actual occurrence of IGP among natural enemies is the basis for the improvement of the biological control effect in the field.

Among the natural enemies of aphids, it is common for ladybeetles to prey on parasitoids, which grow in the host until adult emergence. During this process, the host aphid gradually turns into a mummy, which is easy to be consumed by ladybeetles. Evidence for the occurrence of IGP usually comes from direct observation or feeding selection experiments [5,6,7], but it is difficult to disentangle the relationships between different trophic levels of species when the food web contains multiple arthropods. Therefore, molecular methods based on diagnostic PCR technology have been widely used to detect aphid–parasitoid, aphid–predator and parasitoid–predator interactions [8,9,10,11]. A multiplex system allows the amplification of more than one targeted DNA fragment through a combination of primer pairs [12,13,14]. However, few studies have tracked complete trophic interactions and are limited to aphid–parasitoid or aphid–predator interactions.

With the succession of cotton pests in Xinjiang, aphids have become a major pest affecting cotton yield in recent years [15]. The rampant rise of *Aphis gossypii* Glover and other aphids has caused a serious loss of cotton yield [16]. Ladybeetles and parasitoids are dominant species in the natural enemy community of aphids in local cotton fields [17]. The predatory ladybeetle community mainly includes *Hippodamia variegata* (Goeze), *Propylaea quatuordecimpunctata* (Linnaeus), *Oenopia conglobata* (Linnaeus), *Coccinella tredecimpunctata* Linnaeus, and *Stethorus punctillum* Weise. Survey results of field population density showed that *H. variegata* was abundant in cotton fields in many areas of Xinjiang [18,19,20], and was a dominant predator [21,22]. Regarding aphid parasitoids in the cotton fields of Xinjiang, the population dynamics and parasitism rate of aphids have been investigated and the composition of the aphid parasitoid community has been determined (65% primary parasitoids, 35% hyperparasitoids). Among the four primary parasitoid species, the proportion of *Binodoxys communis* (Gahan) accounted for 95.19% [23]. However, up to now, there have been no reports of predation between *H. variegata* and aphid parasitoids.

In a previous study, a multiplex PCR detection system of common aphids, primary parasitoids and hyperparasitoids from a cotton ecosystem in Xinjiang was successfully constructed and applied to the identification of parasitoids within mummies collected in cotton fields [24]. In the present study, food webs of aphids–primary parasitoids–hyperparasitoids–*H. variegata* in the cotton fields of Korla, Xinjiang, were constructed by using the above detection system in combination with field investigation and feeding selection. Firstly, in the laboratory, we compared the effect of aphid density on IGP between *H. variegata* and mummies under three different aphid densities. Secondly, we investigated and recorded the population dynamics of mummies in the cotton fields of Korla. Finally, multiplex PCR was used to detect the predation frequency of ladybeetles on various aphids, primary parasitoids and hyperparasitoids. Quantitative food webs of aphids–primary parasitoids–hyperparasitoids–*H. variegata* were constructed, and the relative abundance of different prey at the same trophic level was evaluated. This study can help in better understanding the trophic relationships among ladybeetles, parasitoids and aphids. With these data, we can devise better plans to use both natural enemies together to control aphids.

## 2. Materials and Methods

### 2.1. Insect Rearing

*A. gossypii*, *B. communis* and *H. variegata* were collected from cotton fields at the Korla Experimental Station, Chinese Academy of Agricultural Sciences (CAAS; 41.75° N, 85.81° E). Next, they were reared at the Langfang Experimental Station, Chinese Academy of Agricultural Sciences (CAAS; 39.51° N, 116.61° E) (Langfang, Hebei Province). *A. gossypii* was reared on young cotton leaves and maintained within screened cages (55 × 35 × 50 cm). *H. variegata* was fed on *Myzus persicae* (Sulzer) in plastic containers (diameter: 8 cm; height: 11.5 cm). *B. communis* was provided with 10% honey in plexiglass rearing cages (30 × 30 × 25 cm), using *A. gossypii* as the host. After the population had stabilized (about 2 weeks), these insects were used for experiments.

### 2.2. Feeding Selection

*H. variegata* adults, 0–24 h after eclosion, were starved for 12 h (water provided) in a Petri dish (diameter: 9 cm; height: 2 cm). Ten newly formed mummies were gently selected with a brush from the plexiglass rearing cages and placed on a new Petri dish that was already covered with a cotton leaf. Next, 0, 10 or 100 3rd-4th-instar *A. gossypii* nymphs were added to the Petri dish. The aphids and mummies were randomly distributed. Each Petri dish was connected with a ladybeetle that had been hungry and was wrapped with a sealing film to prevent aphids from climbing out. The sealing film was pierced with an insect needle for air permeability. After feeding for 4 h, the ladybeetles were removed, and the number of aphids and mummies was recorded. Each treatment was replicated 15 times. All of the tests were completed within a controlled climate chamber (RXZ500D, Ningbo Jiangnan Instrument Factory, Ningbo, China) at 26 ± 1 °C, 70 ± 5% RH and 16:8 h (L:D) photoperiod.

### 2.3. Population Dynamics Investigation and Sample Collection

The field work was carried out on extensively planted cotton fields in Korla, Xinjiang Uygur Autonomous Region. Three monocropping cotton plots with the same growth stage, area and management were selected. From 2017 to 2019, we visually assessed abundance levels of mummified aphids on 50 randomly chosen plants per plot. From June to August of every year, sampling was conducted at seven-day intervals with a population survey. About 30 *H. variegata* adults were collected per plot on each sampling date. Meanwhile, nontarget arthropods (Table S1) were collected for cross-reactivity tests. All samples were individualized in 1.5 mL centrifuge tubes (Axygen, Union City, CA, USA) and immediately stored in 95% ethanol at −20 °C until DNA extraction. Neither insecticide nor herbicide was used on the cotton fields.

### 2.4. Molecular Detection

DNA extraction of both *H. variegata* adults and nontarget arthropods was performed as follows. After cleaning the body surface with ddH_2_O, the ladybeetles collected above were transferred to a new 1.5 mL centrifuge tube individually with tweezers. The tweezers should be soaked in anhydrous ethanol and burned on an alcohol lamp each time, avoiding cross-contamination between samples. The centrifuge tube was placed in liquid nitrogen and cryogenically frozen for 2 min. Next, the whole sample was ground to powder and placed at room temperature. DNA extraction was carried out using a TIANapm Genomic DNA Kit (TIANGEN, Beijing, China) following the manufacturer’s instructions. Finally, 50 μL TE buffer was added for elution, and the DNA products were stored at −20 °C.

Three multiplex PCR diagnostic systems and one singleplex PCR system were developed and used for the rapid and accurate identification of four aphid, four parasitoid and seven hyperparasitoid species in Xinjiang cotton fields. The target species and corresponding band sizes [24] are shown in Table 1.

DNA samples of *H. variegata* extracted above were amplified following the procedures described in Li et al. [24]. The negative controls were ddH_2_O and *H. variegata* adults which were starved for 24 h, while the positive controls were target insects. A set of DNA samples from nontarget species from the Korla cotton fields (Table S1) were analyzed to test the specificity of the system.

Amplified DNA fragments were separated on 2% agarose gels and visualized using a gel imaging system (UVITEC Essential V6, Cambridge, UK).

### 2.5. Data Analysis

Under different densities of *A. gossypii*, the differences in consumption of mummies were compared via one-way ANOVA, and then multiple comparisons were conducted with Tukey’s HSD test at the 0.05 level. The difference in consumption of aphids was analyzed using a *t*-test. In particular, the difference in consumption between aphids and mummies was explored using a *t*-test when they had the same density (10:10). All the data were statistically processed using Excel 2016 and SPSS25.0. The food web diagram was drawn using the “Igraph” package of R 4.0.5 soft, and the others were drawn using Graphpad Prism 8 software.

## 3. Results

### 3.1. Feeding Selection of Live and Mummified A. gossypii by H. variegata

**Figure 1:**
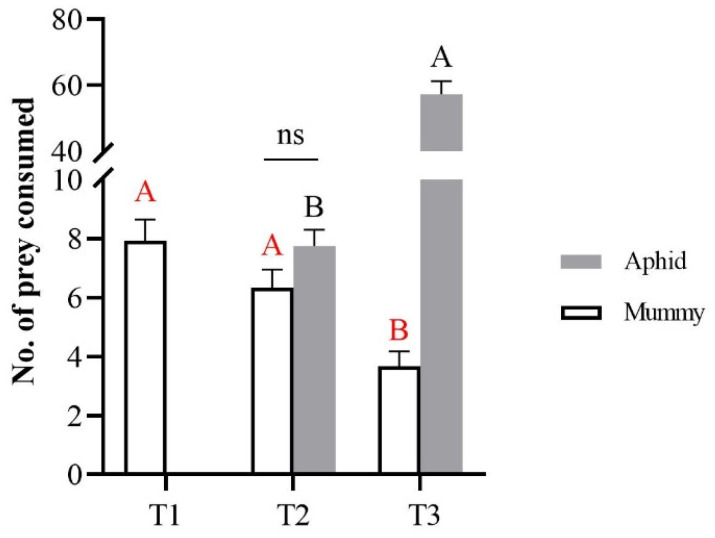
The number of prey (mean ± SE) consumed by *Hippodamia variegata* adults after four hours. T1: 10 mummies and 0 aphids; T2: 10 mummies and 10 aphids; T3: 10 mummies and 100 aphids. Different letters above columns indicate significant differences in mummy consumption under different aphid densities, “A, B” of red color indicate a comparison between consumption of the mummies and “A, B” of black color indicate a comparison between consumption of the aphids (*p* < 0.01, Tukey’s HSD test); “ns” denotes no significant difference between the consumption of aphids and mummies with 10 aphids and 10 mummies per dish (*p* > 0.05).

The experimental results showed that, despite the change in density of *A. gossypii*, *H. variegata* preyed on the mummies (Figure 1). As the aphid density increased, mummy consumption by *H. variegata* clearly decreased (*F* = 12.22; *df =* 2, 22; *p* < 0.001). Compared to the control with no aphids, the predation of mummies decreased by 20.17% and 53.78% in the other two treatments (10 aphids or 100 aphids, respectively). When the density of aphids increased from 10 to 100 aphids per dish, there was a significant increase in the number of consumed aphids (*t* = −11.77, *df* = 14.56, *p* < 0.001). However, there was no significant difference in consumption between the aphids and mummies when they had the same density (*t* = −1.53, *df* = 28, *p* = 0.139).

### 3.2. Dynamics of Mummies in the Cotton Field

**Figure 2:**
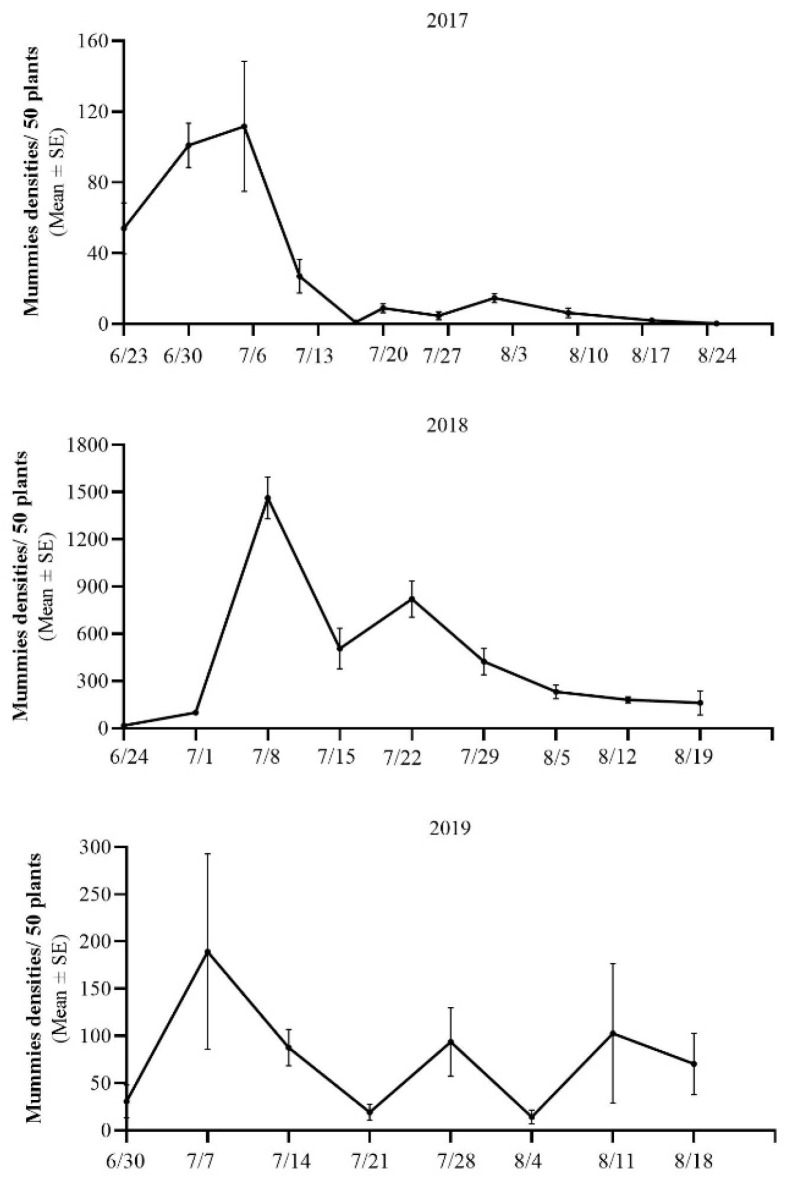
Dynamics of the mummy population in the cotton fields from 2017 to 2019.

Across the three study years (Figure 2), the dynamics of the mummy population recorded in the cotton fields were basically consistent. The mummies appeared on cotton seedlings in late June, with the number increasing gradually and peaking in early July. There was a secondary peak in late July, and then the mummy population decreased, eventually disappearing. However, the density of the mummies varied greatly over the 3 years (2017: 111.67 ± 36.67; 2018: 1463.33 ± 132.77; 2019: 189 ± 103.59 individuals per 50 plants at the peak).

### 3.3. Molecular Detection of Filed-Collected H. variegata

#### 3.3.1. Detection Rate of Aphids, Primary Parasitoids and Hyperparasitoids

The three multiplex PCR detection systems and singleplex PCR detection system could successfully amplify the DNA of the corresponding target prey with high specificity (Figures S1–S4). A total of 2090 ladybeetles collected in the Korla cotton fields from 2017 to 2019 were detected using the above system, and 398 of them were negative to all detection targets (2017: 255; 2018: 71; 2019: 72 individuals). DNA fragments of aphids, primary parasitoids and hyperparasitoids were detected in all three years. In terms of the detection frequency of different groups, among the 2090 samples, aphids were detected 1713 times, followed by primary parasitoids 933 times and hyperparasitoids only 92 times (Table 2).

**Figure 3:**
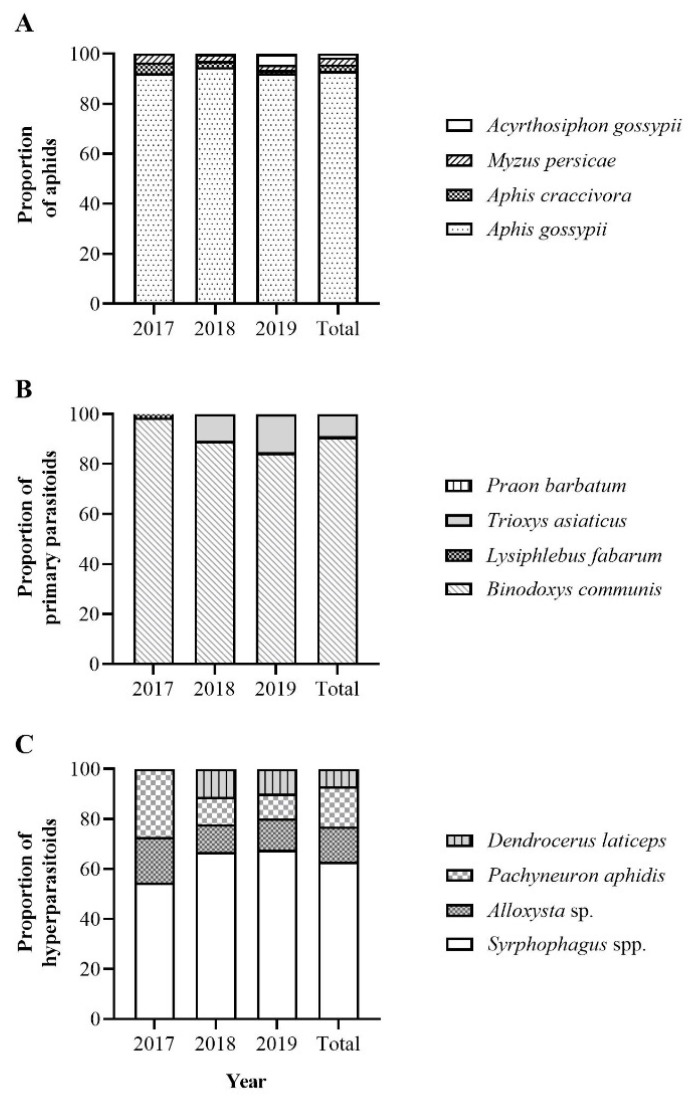
The proportion of aphids (**A**), primary parasitoids (**B**) and hyperparasitoids (**C**) consumed by *Hippodamia variegata* over 2017–2019.

Four aphid species in cMP1 were detected in the ladybeetle samples (*Acyrthosiphon gossypii* Mordviiko, except in 2017). *A. gossypii* was the most abundant species, accounting for more than 90% of the total aphids detected (Figure 3A). Through cMP2, *B. communis* was detected in the largest proportion of samples, which was much higher than the proportion of *Trioxys asiaticus* Telenga, *Lysiphlebus fabarum* (Marshall) and *Praon barbatum* Mackauer over the 3 years. The four primary parasitoid species were detected in 2019, while only two of the primary parasitoid species (2017: *B. communis* and *L. fabarum*; 2018: *B. communis* and *T. asiaticus*) were detected in 2017 and 2018 (Figure 3B). Seven hyperparasitoid species contained in cMP3 and cSP1 had also been detected, but at a lower detection rate. *Syrphophagus* spp. (three species: *Syrphophagus aphidivorus*, *Syrphophagus* sp., *Syrphophagus taeniatus*) dominated the seven hyperparasitoid species, varying between 54.55% and 67.61%. The proportions of the remaining species from high to low were *Pachyneuron aphidis* (Bouché)*, Alloxysta* sp. and *Dendrocerus laticeps* (Hedicke) (Figure 3C). However, *Asaphes suspensus* (Nees) was not detected in all samples.

According to the average percentage of different groups detected in the three years (Figure 3), *A. gossypii* was the main species, accounting for 93.08% of the total detected amounts of aphids, followed by *M. persicae*, *Aphis craccivora* Koch and *Ac. gossypii*, accounting for 2.72%, 2.72% and 1.48%, respectively. The proportions of primary parasitoids detected were as follows: *B. communis* 90.76%, *T. asiaticus* 8.67%, *L. fabarum* 0.57% and *P. barbatum* 0.08%. The proportions of the seven hyperparasitoids detected from high to low were *Syrphophagus* spp. (62.94%), *P. aphidis* (16.08%), *Alloxysta* sp. (13.99%), *D. laticeps* (6.99%) and *A. suspensus* (0%).

#### 3.3.2. Quantitative Food Webs of Aphids–Primary Parasitoids–Hyperparasitoids–*H. variegata*

**Figure 4:**
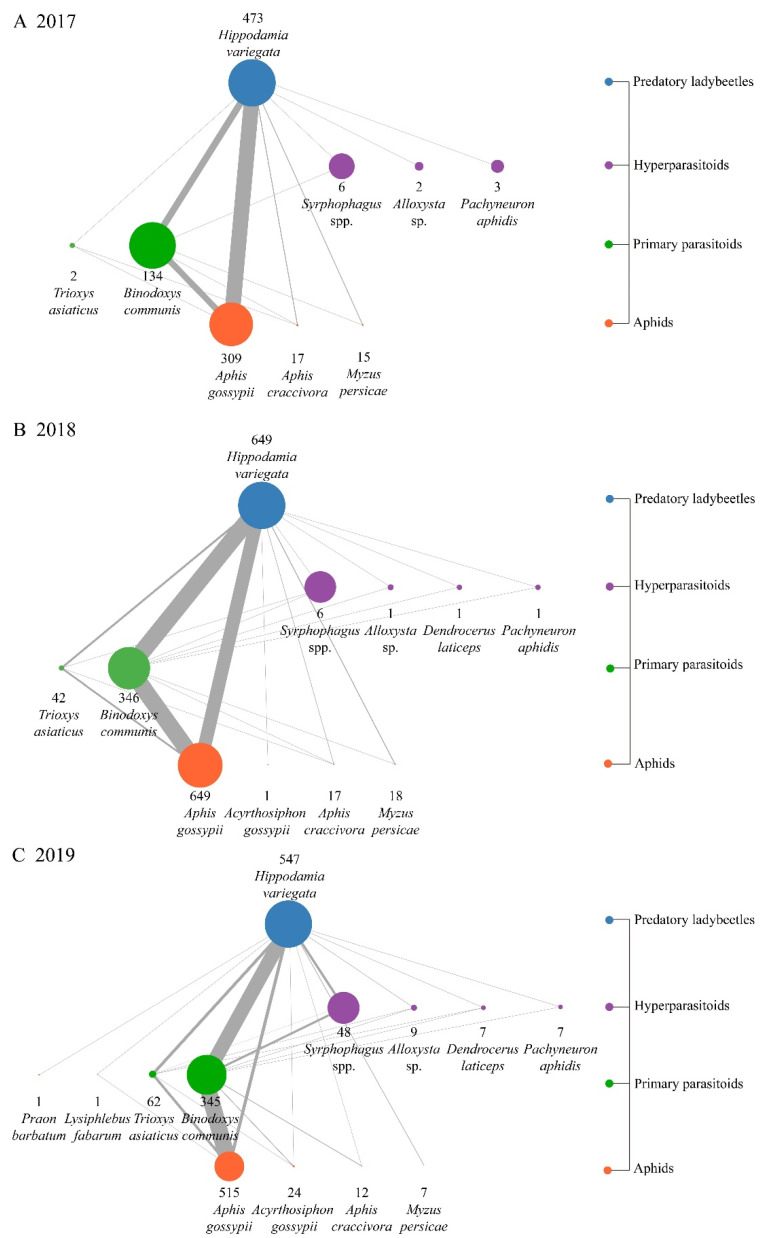
Quantitative food webs of aphids, primary parasitoids, hyperparasitoids and *Hippodamia variegata* in cotton fields. Each circle represents a species; the number below the circle is the detection frequency of the species, and the size of the circle represents the relative abundance of the species at the same trophic level. The width of the connecting lines represents the relative number of higher trophic species attacking the lower trophic species.

The samples with positive amplification were screened from all the ladybeetles, and then, food webs were constructed according to the detection results (Figure 4). The proportions of aphids and parasitoids that were predated on together by the ladybeetles increased year by year, and the combination of *A. gossypii* and *B. communis* dominated, followed by the combination of *A. gossypii* and *T. asiaticus*. Only few hyperparasitoids were simultaneously detected. A total of 6.14% of ladybeetles tested positive for *A. gossypii*, *B. communis* and *Syrphophagus* spp. together in 2019, while less than 1% of the samples tested positive for other combinations.

## 4. Discussion

Intraguild predation is a complex interspecific relationship. In this study, the predation relationship between *H. variegata* and mummies with different densities of aphids was investigated. The results showed that, when the density of aphids increased from 10 to 100 aphids per dish, there was a significant increase in the number of consumed aphids. When the experimental treatment was 10 aphids and 10 mummies per dish, there was no obvious preference for live aphids or mummies. As the aphid density increased to 100 per dish, the consumption of mummies decreased significantly. In conclusion, following an increasing density of live aphids, the predator showed an obvious preference for live aphids rather than mummies, but when there was a low prey density, there was no significant preference between live aphids and mummies (i.e., 10 mummies + 10 live aphids in the T2 treatment). Actually, the parasitism rate of aphids was relatively low (less than 10%) in a previous survey [25]. Therefore, the ladybeetles preyed primarily on live aphids as natural enemies, supplemented by IGP on mummies.

The mummies were concentrated in July and decreased significantly in August. The population reached the highest peak in early July, followed by a secondary peak in middle or late July, and gradually declined. The survey of Li et al. [26] in the cotton fields of southern Xinjiang showed that the peak population of cotton aphids in the field was usually from late June to early July. Li et al. [23] reported the dynamics of cotton aphid populations in Korla, which reached a peak in mid-June and then gradually declined, and the parasitism rate increased with the increase in aphids, but lagged behind aphids. Following the population dynamics and host preferences of various predatory natural enemies in northern Xinjiang, Yang [27] found that *H. variegata* appeared in the cotton field in mid-June, and then reached a peak over late June to early July. A second peak occurred in late July, and then the population decreased. The density of *H. variegata* was significantly positively correlated with the aphid population. In conclusion, the spatio-temporal coincidence of the peak and dynamics of *A. gossypii*, mummies and *H. variegata* in the cotton fields provided convenient conditions for the occurrence of IGP.

In this study, the relative predation rates of several common aphids, primary parasitoids and hyperparasitoids in *H. variegata* were systematically detected for the first time. In addition, there were 398 ladybeetles with negative responses to all the tested targets. The possible reason is that the collection time was too long since the ladybeetles last fed, and the prey DNA in the gut had been digested and degraded. Rondoni et al. [13] found that the DNA detection of prey in the gut of *Harmonia axyridis* (Pallas) larvae decreased with the increase in feeding time, and the digestive half-life of *Eucallipterus tiliae* (Linnaeus) after feeding was 3.1 h. Otherwise, *H. variegata* has a wide range of prey, not only preying on a variety of insects pests [28], but also using pollen as a nutritional supplement [29]. Liang et al. found that, by providing pollen as a supplementary food, the IGP between *H. axyridis* and *Propylea japonica* (Thunberg), two predators of aphids, could be reduced [30]. Therefore, some samples collected in the field may not have been fed on aphids or parasitoids.

In the field, ladybeetles preyed on parasitoids mainly through the parasitized aphids, including live and mummified aphids containing parasitoids. DNA fragments of both aphids and parasitoids were often detected in ladybeetles simultaneously. In the samples that consumed parasitoids, 85.02% of them were positive for aphids and primary parasitoids simultaneously, 6.94% were positive for aphids, primary parasitoids and hyperparasitoid and only 6.39% were positive for parasitoids alone, which were directly preyed on by ladybeetles. Direct predation on parasitoids by predators has already been reported. Traugott et al. [31] used a combination of singleplex PCR and multiplex PCR to track the nutritional interactions among various predators, aphids and parasitoids in a winter wheat field in Warwickshire, UK, and found that 7.5% of predators captured at the end of May had eaten aphid parasitoids. More than 50% of the parasitoids’ DNA came from direct predation, and there were significant differences between predator taxa at levels at which the predators feed on pests and parasitoids, indicating that the species of the predator itself greatly influenced the direct or indirect feeding of parasitoids. Diagnostic PCR technology based on specific primer amplification has become a common method to study the trophic interactions among arthropods [32,33,34], especially in open habitats with complex trophic interactions. For example, Lenka et al. [35] detected the DNA of natural predators Anyphaena and Philodromus in orchards, and found that the frequency of the two kinds of spider species’ predation on pear psylla was much higher than that of IGP. Ortiz-Martine et al. [10] revealed the frequency of IGP among ladybeetles and parasitoids in wheat fields under different landscape backgrounds. The results showed that the complexity of the landscape background did not affect the IGP and population dynamics of aphids in the wheat fields. However, the technology above still has its limitations, as the detection results can only determine whether predation occurs, but cannot effectively assess the prey amount. Paula et al. [36] determined the DNA sequence in the gut of natural enemies through DNA shotgun sequencing, and then compared it with the mitochondrial gene bank to determine the prey species. Combined with the bioinformation technology, they estimated the prey consumption through the reverse regression simulation of the detected prey readings. However, this method has a high cost and few genome sequence databases for reference, so it is necessary to improve the sequence database continuously in further study.

Trophic webs can quantitatively describe the basic structure and intensity of species interactions [37,38,39]. The construction of arthropod food webs is mainly based on the systematic analysis of a large number of samples collected in the field, so as to obtain the trophic interactions between predators and prey, parasites and hosts, etc. [40,41]. In the quantitative food web of predatory arthropods, “quantitative” refers to the occurrence frequency of the trophic links between different predators and prey [32]. In this study, the DNA extracts of *H. variegata* collected in the field were used as the template for detection by three multiplex PCRs and a singleplex PCR. Based on the results, three quantitative food webs of aphids, primary parasitoids, hyperparasitoids and ladybeetles over 2017–2019 were established. In order to reduce the interference of false positive amplification, a variety of nontarget arthropods’ DNA was extracted to determine the specificity of the system. The results showed that *H. variegata* preyed on four types of aphids, four primary parasitoids and six hyperparasitoids. *A. gossypii*, as the dominant species of aphid, interacted with three primary parasitoids: *B. communis*, *T. asiaticus* and *L. fabarum*. *B. communis*, as the primary parasitoid with the highest detection rate, not only interacted with all four aphid species in the detection system, but also interacted with all six hyperparasitoids. The detection rate of *T. asiaticus* was second only to that of *B. communis*. It interacted with three aphid species: *A. gossypii*, *A. craccivora* and *Ac. gossypii*, as well as five hyperparasitoids, but not *P. aphidis*. In this paper, the width of the connecting lines in the food webs represents the relative frequency of attacks of the higher-trophic-level species on the lower. The parasitoid populations predated on by the ladybeetle are abundant, but the detection frequency of *A. gossypii* in combination with *B. communis* was much higher than that of other species, which is highly consistent with the species composition of the aphids and parasitoids in the cotton fields of Xinjiang [23].

Although parasitoids are important natural enemies to control aphid population growth in Xinjiang cotton fields, the control effect may be impaired by IGP with predators. Therefore, it is necessary to systematically evaluate the intraguild predation effect in the food web. In this study, IGP between *H. variegata* and parasitoids was demonstrated. The population dynamics of mummies in the Korla cotton fields were investigated and recorded, and the composition ratio of aphids and parasitoids, which are preyed on by the dominant natural enemy of ladybeetles in cotton fields, was revealed by molecular detection. Quantitative food webs were constructed to describe the trophic interactions among different aphids, primary parasitoids, hyperparasitoids and *H. variegata* and their relative frequencies of occurrence. The above information will help to evaluate the pest control ability of *H. variegata* more comprehensively and strengthen the strategies for the biological control of aphids.

## Figures and Tables

**Table 1:** Amplified DNA fragment sizes of aphids, primary parasitoids and hyperparasitoids.

System	Species	Product Size/bp
cMP1	*Acyrthosiphon gossypii* Mordviiko	149
	*Aphis gossypii* Glover	191
	*Aphis craccivora* Koch	291
	*Myzus persicae* (Sulzer)	469
cPriMP2	*Binodoxys communis* (Gahan)	233
	*Lysiphlebus fabarum* (Marshall)	322
	*Trioxys asiaticus* Telenga	453
	*Praon barbatum* Mackauer	164
cHypMP3	*Dendrocerus laticeps* (Hedicke)	534
	*Alloxysta* sp.	362
	*Syrphophagus* spp.	425
	*Pachyneuron aphidis* (Bouché)	216
cSP1	*Asaphes suspensus* (Nees)	163

**Table 2:** Detection frequency of aphids, primary parasitoids and hyperparasitoids in field-collected *Hippodamia variegata* individuals.

Year	Ladybeetles Tested * No.	Detection Frequency		
		Aphids	Primary Parasitoids	Hyperparasitoids
2017	728	474	136	11
2018	743	683	387	10
2019	619	556	410	71
Total	2090	1713	933	92

* An individual of *H. variegata* may be detected for more than one species of prey.

## Data Availability

All the data analyzed in this study are included in this article.

## Supplementary Materials

The following supporting information can be downloaded at: https://www.mdpi.com/2075-4450/14/1/81/s1. Table S1: Nontarget arthropods of cotton field for primers species-specific detection; Figure S1: Specificity and efficacy of multiplex PCR system MP1 for aphid detection; Figure S2: Specificity and efficacy of multiplex PCR system MP2 for primary parasitoids detection; Figure S3: Specificity and efficacy of multiplex PCR system MP3 for hyperparasitoid detection; Figure S4: Specificity and efficacy of multiplex PCR system SP1 for hyperparasitoid detection.
