# Supplementary material for: Intraguild Predation of Hippodamia variegata on Aphid Mummies in Cotton Field
Source: Insects. 2023 Jan 13;14(1):81. doi: 10.3390/insects14010081 (PMC9862560; doi:10.3390/insects14010081)
Supplement: Supplementary file 1 [file insects-14-00081-s001.zip › insects-2117809-supplementary.pdf]

Supplementary Table and Figures

Table S1. Nontarget arthropods of cotton field for primers species-specific detection.

Order	Family	Species
Coleoptera	Coccinellidae	*Hippodamia variegata* (Goeze)
		Coccinella undecimpunctata Linnaeus
		Stethorus punctillum Weise
Diptera	Syrphidae	*Eupeodes corollae* (Fabricius)
		Episyrphus balteatus (De Geer)
Hymenoptera	Braconidae	*Binodoxys communis* (Gahan)
Neuroptera	Chrysopidae	*Chrysoperla sinica* (Tjeder)
Hemiptera	Miridae	*Deraeocoris punctulatus* (Fallen)
		Campylomma verbasci (Meyer-Dür)
		Adelphocoris lineolatus (Goeze)
		Lygus pratensis (Linnaeus)
Lepidoptera	Noctuidae	*Helicoverpa armigera* (Hübner)
		Spodoptera exigua (Hübner)
		Agrotis segetum (Denis & Schiffermüller)
		Agrotis c-nigrum (Linnaeus)
		Agrotis exclamationis (Linnaeus)
Thysanoptera	Thripidae	*Frankliniella intonsa* (Trybom)
Acari	Tetranychidae	*Tetranychus urticae* Koch
		Tetranychus turkestani (Ugarov & Nikolskii)

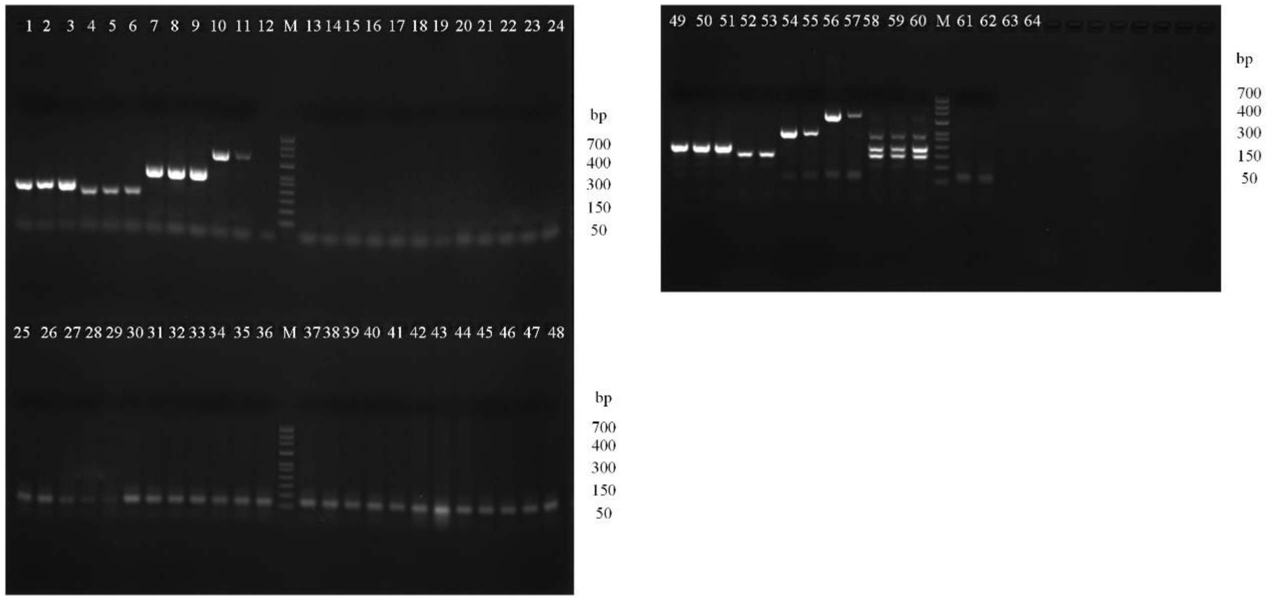

Figure S1. Specificity and efficacy of multiplex PCR system MP1 for aphid detection. 1-3: *Aphis gossypii* Glover, 4-6: *Acyrthosiphon gossypii* Mordvilko, 7-9: *Aphis craccivora* Koch, 10-11: *Myzus persicae* (Sulzer), 12-14: ddH_2O, 15-16: *Hippodamia variegata* (Goeze), 17-18: *Coccinella undecimpunctata* Linnaeus, 19-20: *Stethorus punctillum* Weise, 21-22: *Syrphus corollae* (Fabricius), 23-24: *Episyrphus balteatus* (De Geer), 25-26: *Campylomma verbasci* (Meyer-Dur), 27-28: *Chrysoperla sinica* (Tjeder), 29-30: *Lygus pratensis* (Linnaeus), 31-32: *Adelphocoris lineolatus* (Goeze), 33-34: *Helicoverpa armigera* (Hübner), 35-36: *Spodoptera exigua* (Hübner), 37-38: *Agrotis segetum* (Denis & Schiffermüller), 39-40: *Agrotis c-nigrum* (Linnaeus), 41-42: *Agrotis exclamationis* (Linnaeus), 43-44: *Frankliniella intonsa* (Trybom), 45-46: *Tetranychus turkestani* (Ugarov & Nikolskii), 47-48: *Episyrphus balteatus* (De Geer), 49-51: *Aphis gossypii* Glover, 52-53: *Acyrthosiphon gossypii* Mordvilko, 54-55: *Aphis craccivora* Koch, 56-57: *Myzus persicae* (Sulzer), 58-60: DNA mix of the four aphids species, 61-62: ddH_2O.

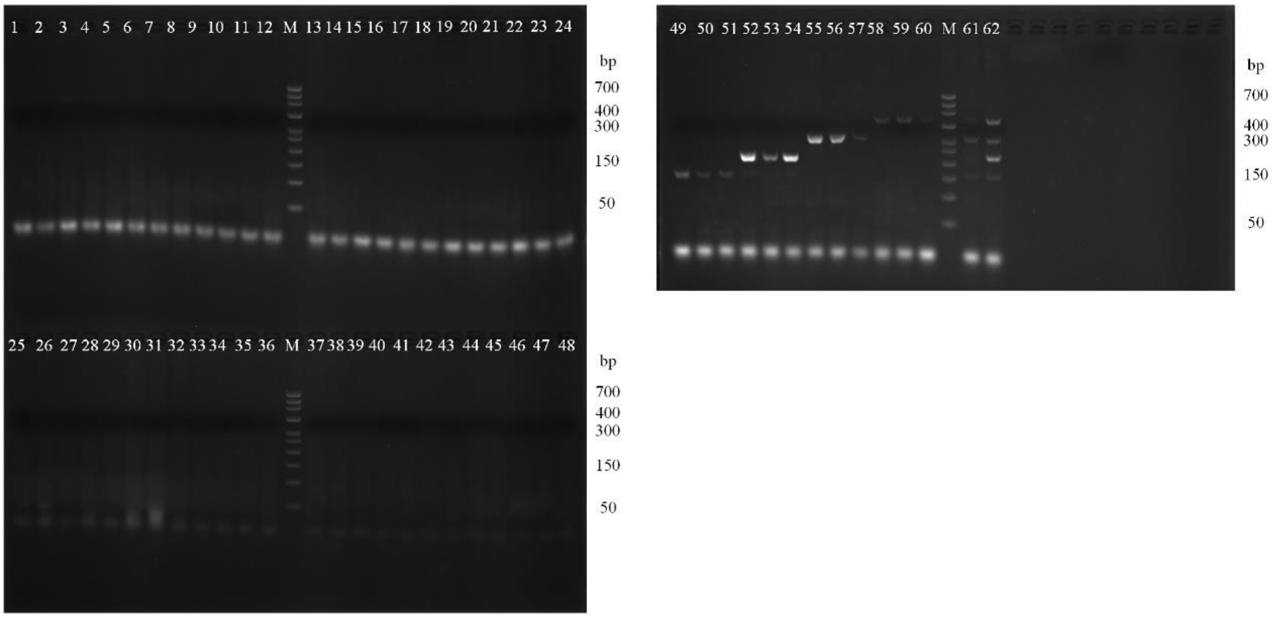

Figure S2. Specificity and efficacy of multiplex PCR system MP2 for primary parasitoids detection. 1-3: *Aphis gossypii* Glover, 4-6: *Acyrthosiphon gossypii* Mordvilko, 7-9: *Aphis craccivora* Koch, 10-11: *Myzus persicae* (Sulzer), 12-14: ddH_2O, 15-16: *Hippodamia variegata* (Goeze), 17-18: *Coccinella undecimpunctata* Linnaeus, 19-20: *Stethorus punctillum* Weise, 21-22: *Syrphus corollae* (Fabricius), 23-24: *Episyrphus balteatus* (De Geer), 25-26: *Campylomma verbasci* (Meyer-Dur), 27-28: *Chrysoperla sinica* (Tjeder), 29-30: *Lygus pratensis* (Linnaeus), 31-32: *Adelphocoris lineolatus* (Goeze), 33-34: *Helicoverpa armigera* (Hübner), 35-36: *Spodoptera exigua* (Hübner), 37-38: *Agrotis segetum* (Denis & Schiffermüller), 39-40: *Agrotis c-nigrum* (Linnaeus), 41-42: *Agrotis exclamationis* (Linnaeus), 43-44: *Frankliniella intonsa* (Trybom), 45-46: *Tetranychus turkestani* (Ugarov & Nikolskii), 47-48: *Episyrphus balteatus* (De Geer), 49-51: *Praon barbatum* Mackauer, 52-54: *Binodoxys communis* (Gahan), 55-57: *Lysiphlebus fabarum* (Marshall), 58-60: *Trioxys asiaticus* Telenga, 61-62: DNA mix of the four primary parasitoids.

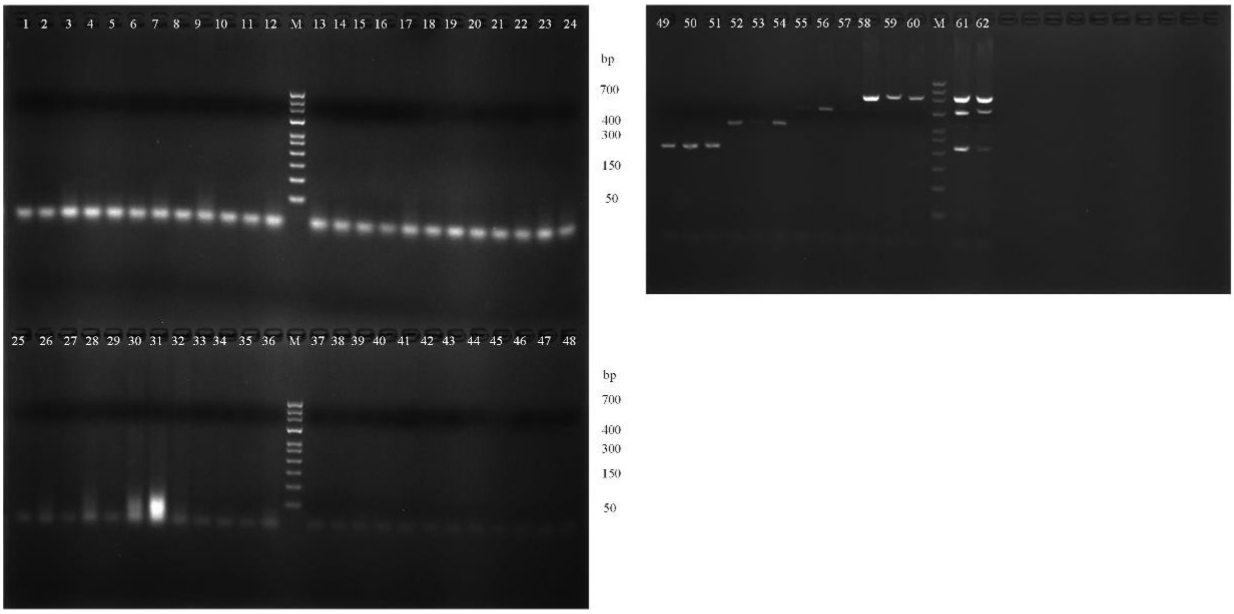

Figure S3. Specificity and efficacy of multiplex PCR system MP3 for hyperparasitoid detection. 1-3: *Aphis gossypii* Glover, 4-6: *Acyrthosiphon gossypii* Mordvilko, 7-9: *Aphis craccivora* Koch, 10-11: *Myzus persicae* (Sulzer), 12-14: ddH_2O, 15-16: *Hippodamia variegata* (Goeze), 17-18: *Coccinella undecimpunctata* Linnaeus, 19-20: *Stethorus punctillum* Weise, 21-22: *Syrphus corollae* (Fabricius), 23-24: *Episyrphus balteatus* (De Geer), 25-26: *Campylomma verbasci* (Meyer-Dur), 27-28: *Chrysoperla sinica* (Tjeder), 29-30: *Lygus pratensis* (Linnaeus), 31-32: *Adelphocoris lineolatus* (Goeze), 33-34: *Helicoverpa armigera* (Hübner), 35-36: *Spodoptera exigua* (Hübner), 37-38: *Agrotis segetum* (Denis & Schiffermüller), 39-40: *Agrotis c-nigrum* (Linnaeus), 41-42: *Agrotis exclamationis* (Linnaeus), 43-44: *Frankliniella intonsa* (Trybom), 45-46: *Tetranychus turkestani* (Ugarov & Nikolskii), 47-48: *Episyrphus balteatus* (De Geer), 49-51: *Pachyneuron aphidi* (Bouché), 52-54: *Alloxysta* sp., 55-57: *Syrphophagus* spp., 58-60: *Dendrocerus laticeps* (Hedicke), 61-62: DNA mix of the four hyperparasitoid.

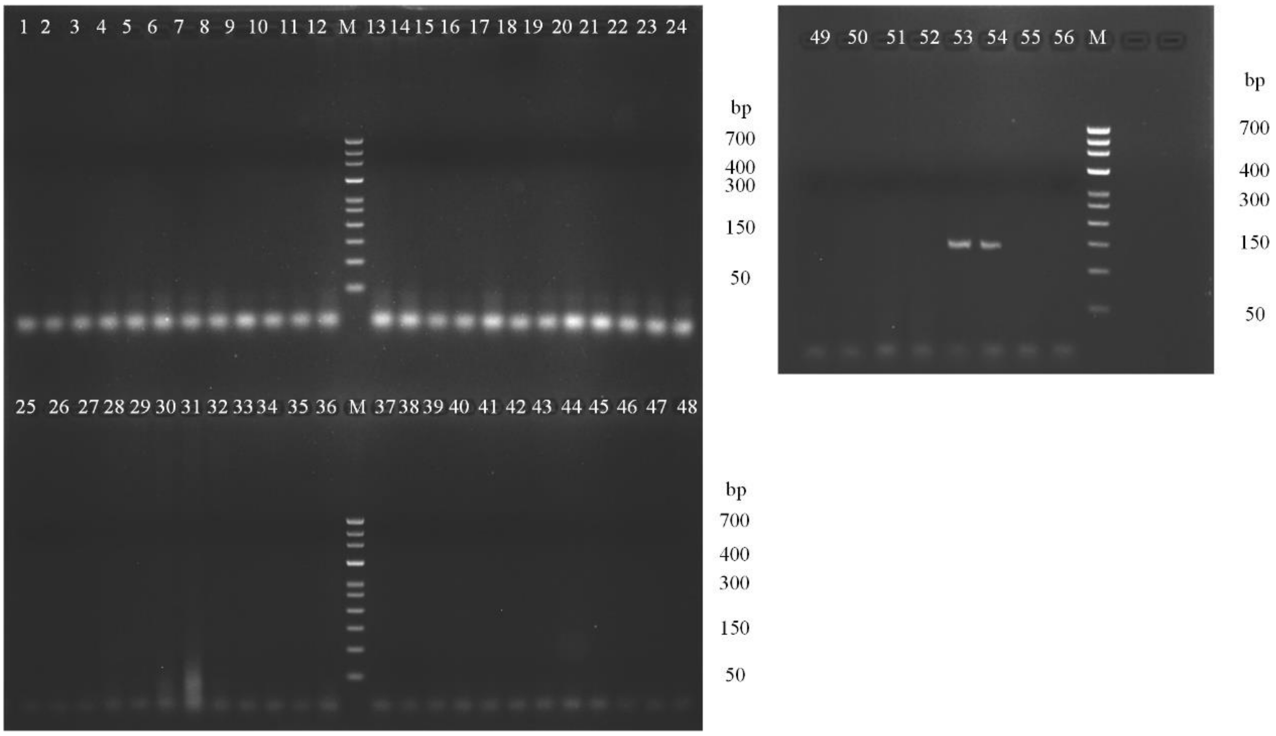

Figure S4. Specificity and efficacy of multiplex PCR system SP1 for hyperparasitoid detection. 1-3: *Aphis gossypii* Glover, 4-6: *Acyrthosiphon gossypii* Mordvilko, 7-9: *Aphis craccivora* Koch, 10-11: *Myzus persicae* (Sulzer), 12-14: ddH$_2$O, 15-16: *Hippodamia variegata* (Goeze), 17-18: *Coccinella undecimpunctata* Linnaeus, 19-20: *Stethorus punctillum* Weise, 21-22: *Syrphus corollae* (Fabricius), 23-24: *Episyrphus balteatus* (De Geer), 25-26: *Campylomma verbasci* (Meyer-Dur), 27-28: *Chrysoperla sinica* (Tjeder), 29-30: *Lygus pratensis* (Linnaeus), 31-32: *Adelphocoris lineolatus* (Goeze), 33-34: *Helicoverpa armigera* (Hübner), 35-36: *Spodoptera exigua* (Hübner), 37-38: *Agrotis segetum* (Denis & Schiffermüller), 39-40: *Agrotis c-nigrum* (Linnaeus), 41-42: *Agrotis exclamationis* (Linnaeus), 43-44: *Frankliniella intonsa* (Trybom), 45-46: *Tetranychus turkestani* (Ugarov & Nikolskii), 47-48: *Episyrphus balteatus* (De Geer), 49-52: *Hippodamia variegata* (Goeze), 53-54: *Asaphes suspensus* (Nees). 55-56: ddH$_2$O.
